# Celiac disease TG2 autoantibodies development: it takes two to tango

**DOI:** 10.1038/s41419-020-2412-5

**Published:** 2020-04-14

**Authors:** Federica Rossin, Mauro Piacentini

**Affiliations:** 1 Department of Biology, University of Rome “Tor Vergata”, Rome, Italy; 2 National Institute for Infectious Disease IRCCS “Lazzaro Spallanzani”, Rome, Italy

**Keywords:** Autoimmunity, Coeliac disease

**Fig. 1:**
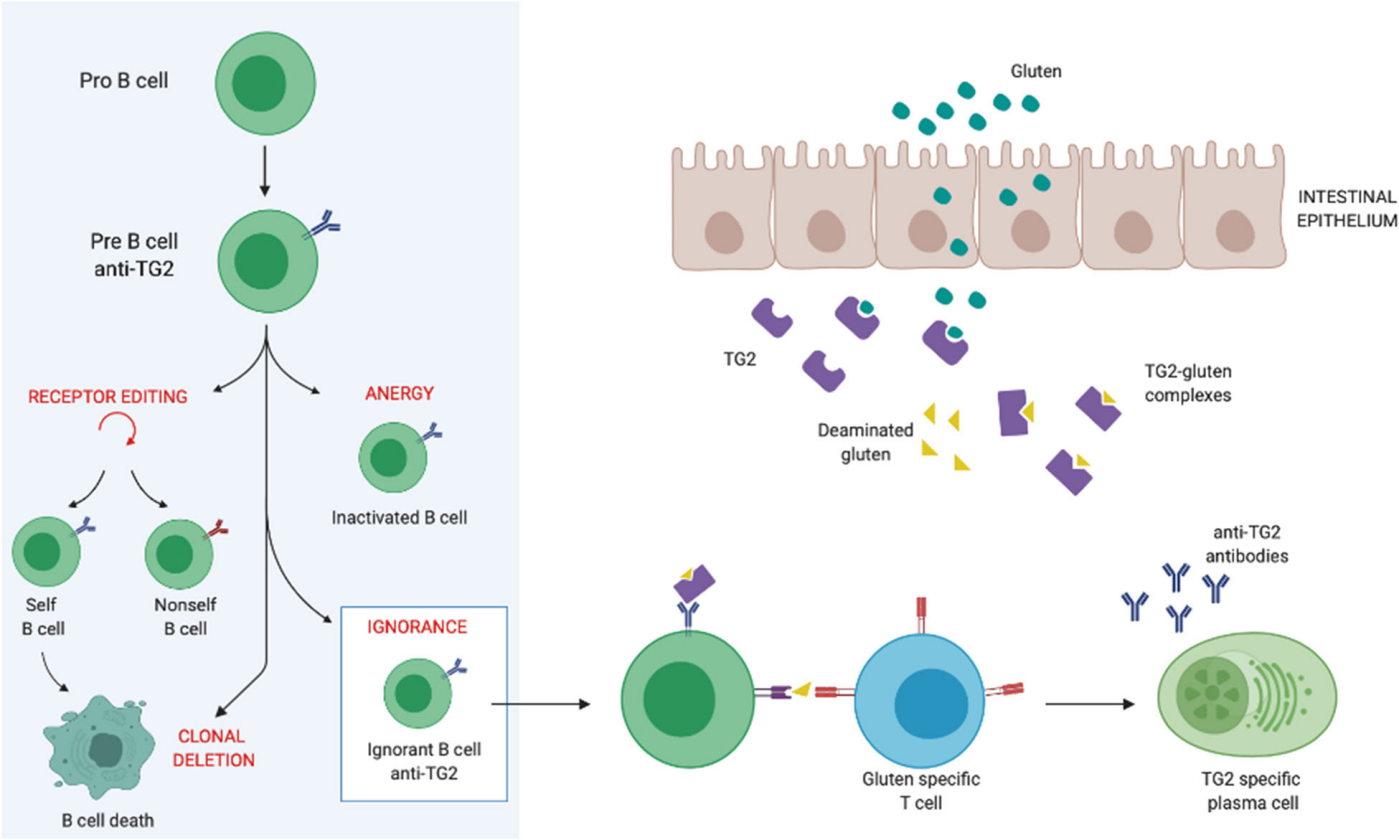
Scheme of TG2 dependent autoantibodies development in CD. Lack of B cell tolerance for TG2 autoreactive B cell leads to the presence of ignorant B cells to TG2. After TG2 catalyzes the deamidation of gluten peptides, TG2–gluten complexes are taken up by TG2-specific B cells that receive help from gluten-reactive T cells becoming producers of autoantibodies.

Celiac disease (CD) is a specific enteropatic response to gluten, a protein found in wheat, barley, and rye. In the people affected by the CD, eating gluten causes an immune response leading to serious changes of small intestine tissue architecture and as a consequence to malabsorption^1,2^. CD is an inflammatory disorder characterized by the leukocyte infiltration that develops in genetically susceptible individuals as the result of an inappropriate immune response to gluten proteins. CD4(+) T cells that recognize deaminated gluten peptides bound to predisposing HLA-DQ molecules (DQ2.5, DQ2.2, and DQ8) play a key pathogenetic role in CD^3^. Interestingly, the deamidation of the gluten peptides is catalyzed by the enzyme type 2 transglutaminase (TG2) to which antibodies are produced representing the hallmark of the disease^4,5^. In addition to autoimmunity, TG2 has been implicated in all major human pathological conditions, including neurodegenerative and metabolic disorders, fibrosis, and cancer. TG2 is a peculiar member of the transglutaminase family since it catalyzes, in addition to the canonical Ca2+-dependent transamidating activity, several other enzymatic activities (GTPase/ATPase, protein disulfide isomerase, protein kinase) as well as non-enzymatic functions based on its non-covalent scaffold interactions with many cellular proteins. Due to its multifunctionality, TG2 has been reported to have a complex biology playing a role in a variety of cellular processes, such as differentiation, survival, apoptosis, autophagy, and cell adhesion^6,7^. The identification of autoantibodies against TG2 in CD was first reported by Dieterich et al. in 1997^8^. Since then, the detection of the IgA anti-TG2 Ab has become the most widely used test both for the diagnosis and initial screening for CD because of its very high sensitivity and specificity^9^. Despite the accepted evidence of the involvement of TG2 in the CD, there are still many open questions about its role in the disease’s pathogenesis. Recently, in addition to the T cells, a role for B cells in the CD pathogenesis is receiving increased attention from the scientific community. A recent study appeared in *Journal Experimental Medicine*^10^ from the Sollid’s group has elucidated the function of B cells in the CD pathogenesis. To address this problem the authors developed an Ig knock-in mouse based on the CD-derived B cell receptor (BCR) able to react with both human and mouse TG2. This mouse was then crossed with *Tgm2* knock out mice producing a new elegant model to study the functional behavior of B cells in the presence and in the absence of the autoantigen. Surprisingly the data obtained by this new animal model showed that there is not clonal deletion and/or anergy development to TG2. Instead, breaking B cell tolerance to the presence of the TG2 autoantigen required the help provided by the T cells. In the *J. Exp. Med.* paper, the authors demonstrated that nontolerized TG2-specific B cells can produce autoantibodies once gluten-specific T cells provide help. In this regard, many studies have proved that the establishment of T cell tolerance is more efficient than that induced in the B cells^11^. Although there is no evidence for TG2 as a target for T cell autoimmunity, the enzyme is able to generate TG2–gluten complexes that the authors showed, by means of a TG2–gluten fusion protein, may indeed stimulate the gluten-specific T cells to provide the required help to ignorant specific B cells (Fig. 1). This type of T cell help has already been shown in other autoimmune diseases whose autoantigens are present at very low concentrations^12^.

Interestingly, the authors explain the absence of tolerance for TG2 by claiming that this “B cell ignorance” is not due to the low affinity of the antigen to the BCR, but to limited antigen availability and thus low degree of receptor occupancy. These findings call into question another challenging aspect related to the localization of TG2 in the extracellular matrix (ECM) under physiological conditions. This is a controversial issue that has debated since 1987 when Upchurch et al.^13^ reported that the enzyme can interacts with fibronectin in the ECM. In following years many studies claim the possibility that TG2 could be present in the ECM under physiological condition interacting with many different protein partners^14^. However, immunohistochemical studies carried out in various organs under physiological conditions did not confirm the presence of extracellular TG2 (ref. ^15^), which instead has been clearly documented in the ECM under pathological conditions such as in kidney and liver fibrosis^16,17^. The paper by Sollid’s group seems to confirm this latter evidence highlighting that the detection of extracellular TG2 is an artifact due to tissue sectioning resulting in the release of the intracellular enzyme. These data are very important to challenge the accepted finding that the TG2 can be localized in the ECM under normal physiological conditions.
